# Small cell neuroendocrine tumour of the endometrium and the importance of pathologic diagnosis

**DOI:** 10.3332/ecancer.2016.668

**Published:** 2016-08-23

**Authors:** Adriana Estruch, Lucas Minig, Carmen Illueca, Ignacio Romero, Jose Luis Guinot, Andrés Poveda

**Affiliations:** Clinical Area of Gynaecologic Oncology, Instituto Valenciano de Oncologia (IVO), Valencia, Spain

**Keywords:** small neuroendocrine tumour, endometrium, pathology diagnosis, endometrial cancer

## Abstract

Small cell carcinoma of the endometrium is a very rare entity. They are very aggressive tumours, with a poor prognosis. They represent a clinical challenge because of a lack of a standardised treatment. We see here a case of a 67-year-old woman with a history of a lobular breast carcinoma, diagnosed in 2002. After presenting with postmenopausal vaginal bleeding in October 2014, she underwent a hysteroscopy-guided biopsy which revealed a metastasis of breast carcinoma. A hysterectomy and bilateral oophorectomy was performed because of uncontrolled uterine bleeding. The pathologic diagnosis was small cell carcinoma (SCC) of the endometrium. A surgical complete cytoreduction was achieved after the case being presented in a multidisciplinary tumour board. Pathologic results revealed metastasis from peritoneal implants of SCC on the endometrium, and metastasis in pelvic and para-aortic lymph nodes from serous carcinoma of the endometrium. A total of four cycles of adjuvant chemotherapy based on cisplatin (80mg/m² day one) and etoposide (100mg/m² day one, two, three) every 21 days was given. The patient experienced persistent disease and died 17 months after the diagnosis. SCC of the endometrium is a very rare and aggressive disease that requires an individualised multidisciplinary management.

## Introduction

Endometrial cancer is the most common tumour affecting the genital organs. Even though endometrioid is the most common histology subtype, other rare histologies have also been described [1]. These rare entities include, for example, small cell carcinoma (SCC) of the endometrium with an estimated incidence of 0.8% of all endometrial carcinomas [2].

Small cell carcinoma is a neuroendocrine tumour which is most commonly diagnosed in the lung [3]. It is estimated that only 5% of those tumours are diagnosed at extrapulmonary organs including gastrointestinal tract and female genital tract [4–5]. SCC of the endometrium is highly aggressive being usually diagnosed at advanced stages of the disease [6]. Because of its rarity, there are few case reports in the literature to date [1], and there is no well-established consensus regarding the histopathology diagnosis and its appropriate treatment. Therefore, here we present a case of SCC of the endometrium with an extensive pathological and immunohistochemical analysis.

## Case report

This is a 67-year-old woman (gravida 2, para 2), with a history of lobular breast carcinoma diagnosed in 2002 (T1N0. estrogen receptor +ve, progesterone receptor +ve, HER-2-ve). She underwent tumourectomy and axillary lymph node dissection. She received whole breast adjuvant radiotherapy (achieving 46 Gy) plus high dose rate brachytherapy (a single session of 7 Gy). Five years of tamoxifen was indicated. The patient was free of relapse up to 2014. On October 2014, she presented with persistent postmenopausal bleeding. A hysteroscopy showed a 10 cm endometrial polyp which was then biopsied. The pathological diagnosis was metastasis of breast carcinoma, possibly lobular histology subtype. A pelvic Magnetic Resonance Imaging (MRI) noted a 100 x 86 mm endometrial heterogenic lesion; with a deep myometrial infiltration reaching the serosa without endocervical involvement. A chest-abdominal-pelvic Computer Tomography (CT) scan revealed no peritoneal implants and no evidence of disease beyond the pelvic mass previously described. Her postmentopausal bleeding persisted, requiring three packets of red blood transfusion. The patient underwent a total hysterectomy with bilateral oophorectomy. A laparoscopy was not performed because of the uterine size and to avoid abdominal contamination. The surgical findings included several 5–10 mm peritoneal implants on the pelvis as well as a uterine tumour infiltrating both ovaries. Surgery with watchful postoperative period was carried out without relevant incidence.

**Figure 1.**
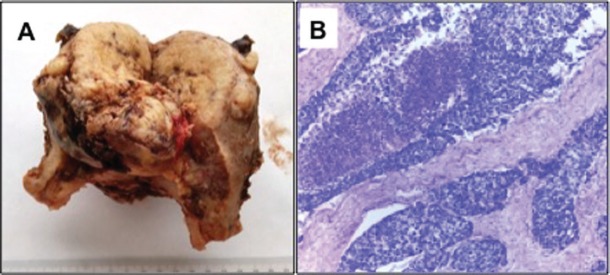
A) Macroscopic tumour: an 8 cm tumour with a myometrial infiltration of more than 50%, reaching the uterine serosa B) H-E 20x: Solid tumoural nests are observed, consisting of small cells with limited cytoplasm.

**Figure 2.**
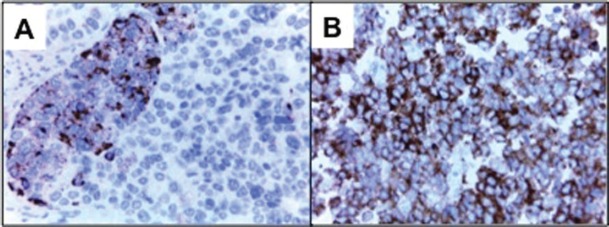
A) Neoplastic nest, consisting of small cells with cytoplasmic positivity to chromogranin A. B) : Tumour cells with cytoplasmic positivity to synaptophysin.

A final pathology diagnosis revealed a high-grade (G3) small cell neuroendocrine tumour of the endometrium. At macroscopy an 8 cm tumour, with a myometrial infiltration of more than 50%, reaching the uterine serosa was observed (Figure 1-A). At microscopy with haematoxilin and eosine, lymphovascular embolisation and parametrial stromal infiltration were observed (Figure 1-B). Immunohistochemistry analysis showed synaptophysin++; CD56 +; chromogranin A+ in isolated cells; Ki-67, 80% (Figure 2).

A cytoreductive surgery was decided in a multidisciplinary tumour board. Surgical findings, 22 days after the initial surgery, revealed pelvic and mesenterium peritoneal implant from 10–40 mm. A radical resection of pelvic peritoneum and mesenteric peritoneal implants along with pelvic and aortic retroperitoneal radical lymphadenectomy were performed achieving a complete tumour debulking.

**Figure 3.**
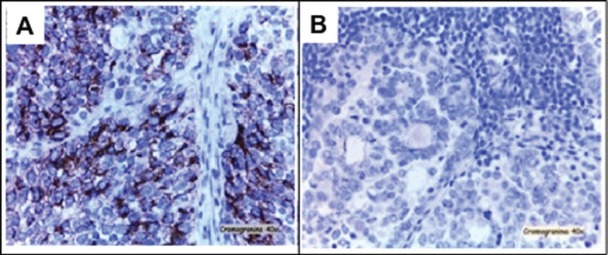
A) Peritoneal implants, consisting of small cells and IHQ pattern very similar to neuroendocrine primary tumour. B) Lymph nodes metastasis from serous carcinoma with synaptophysin (-) and chromogranin A (-).

The pathological diagnosis of the peritoneal implants showed metastasis of neuroendocrine tumour, with similar features at immunohistochemical (IHQ) as previously described (Figure 3-A). However, high-grade serous carcinoma with synaptophisin (-) and chromogranin A (-) was diagnosed in 5 out of 13 pelvic lymph nodes and in 20 out of 25 para-aortic lymph nodes (Figure 3-B). Four cycles of adjuvant chemotherapy based on cisplatin (80mg/m² day one) and etoposide (100mg/m² day one, two, three) every 21 days were given. The patient experienced persistence of the disease and died 17 months after the diagnosis.

## Discussion

Small cell carcinoma (SCC) is a neuroendocrine tumour arising from the neuroendocrine cells in lymph nodes, endocrine glands, skin, and the general endocrine system. SCC is more frequently found in the lung (95% of small cell neuroendocrine tumours) and in the gastrointestinal tract [5].

Tumours that originates from gynaecological organs are, however, extremely uncommon. There are only 80 cases published in the English literature of SCC with its origin on the endometrium [1]. The mean age at diagnosis is over 60 years (range 23–87). As in our case, abnormal or postmenopausal vaginal bleeding is the most frequent symptom. However, it has been associated with different para-neoplastic signs and symptoms such as retinopathy, Cushing’s syndrome, or membranous glomerulonephritis [7].

The present case reports the diagnoses as a mixed tumour with a high-grade neuroendocrine carcinoma in the uterus and with peritoneal implants and metastasis of endometrial high-grade serous carcinoma in the retroperitoneal lymph nodes. The presence of these two components suggests that it might be a mixed tumour in the early origin of tumourgenesis, the neuroendocrine carcinoma joins with the high-grade serous carcinoma.

This is one the most likely interpretation, but there is another explanation also. It is that the primary tumour was endometrial serous carcinoma all along which then dedifferentiated, appearing as undifferentiated neuroendocrine tumour afterwards.

The histology difference between the peritoneal and the lymph nodes metastases can be associated with the pattern of dissemination of neuroendocrine tumours, which normally spread to the peritoneum; and the serous carcinomas which metastasizes usually in the lymphatic system.

The mixed compound of SCC of the endometrium has also been previously described in over 50% of tumours. The most common histology diagnosed ones included adenocarcinoma component, carcinosarcoma, and adenosquamous carcinoma [8–9].

It has been proposed that in cases of mixed tumours, the prognosis is determined by the small cell component [1] given the fact that the metastases are commonly associated with the SCC component. It is interesting to note, however, that in our case both component of the primary tumour were able to metastasise to the retroperitoneal lymph nodes (serous component) and to the peritoneum (SCC component).

The histology diagnostic criteria for SCC have been proposed and include [9] the unmistakable evidence of its endometrial origin. Wiith standard haematoxylin-eosin stained sections, it is observed as a dense sheet-like growth of morphologically similar small/intermediate-sized tumour cells which also has immunohistochemical reactivity for one or more neuroendocrine markers. All of these parameters were found to be fulfilled in our case.

Neuroendocrine markers are essential to establish the definitive diagnosis. The main markers used include chromogranin A, synaptophysin, neurospecific enolase, Leu-7, and CD56 [9–10].

It is estimated that over 60% of SSC of the endometrium are diagnosed at advanced stage (III–IV), [8–9] with a very poor prognosis, even if they are diagnosed at early stages [10].

Given the rarity of this entity, it is difficult to establish a consensus regarding the appropriate management of these tumours. The strategies of treatment used is multimodal, resembling the treatment of this disease when it is present in the lung.

Although there is no evidence that primary radical surgery has an impact in the course of the disease, it is usually the first step in the management of these tumours described in the literature [1]. It includes primary surgical cytoreduction with total hysterectomy, bilateral salpingo-oophorectomy, omentectomy, and pelvic/para-aortic lymphadenectomy, followed by platinum-based chemotherapy and radiotherapy [8–9, 11]. By mimicking the adjuvant treatment used in lung tumours, chemotherapy based on cisplatin and etoposide is generally indicated. However, there have also been other combinations used including docetaxel + cisplatin , paclitaxel + carboplatin, cisplatin + irinotecan [12–13].

Tumour relapse can be as high as 50% for early stage and 88% for advanced stage tumours [6]. A recent review of the literature [1] showed a mean overall survival (OS) of 22 and 12 months for women diagnosed at early stages or at advanced stages respectively. Longer survivors were scarcely described with a mean OS of 3–9 year for early stage and 2–36 months for patients with advanced stage disease [7, 14].

## Conclusion

SCC of the endometrium is a very rare and aggressive disease that requires an individualised multidisciplinary management. Specialist pathologists are crucial to obtain an appropriate histology diagnosis.
